# Body Mass Index Specifiers in Anorexia Nervosa: Anything below the “Extreme”?

**DOI:** 10.3390/jcm11030542

**Published:** 2022-01-21

**Authors:** Federica Toppino, Paola Longo, Matteo Martini, Giovanni Abbate-Daga, Enrica Marzola

**Affiliations:** Eating Disorders Center, Department of Neuroscience “Rita Levi Montalcini”, “Città della Salute e della Scienza” Hospital of Turin, University of Turin, 10126 Turin, Italy; toppinofederica@gmail.com (F.T.); paola.longo@unito.it (P.L.); matteo.martini@unito.it (M.M.); enrica.marzola@unito.it (E.M.)

**Keywords:** eating disorders, anxiety, depression, body image, outcome, hospitalization

## Abstract

The validity of body mass index (BMI) specifiers for anorexia nervosa (AN) has been questioned, but their applicability to inpatients with extremely low BMIs and their prognostic validity are currently unknown. Therefore, we designed this study: (a) to test current BMI specifiers in severe inpatients; (b) to explore a “very extreme” specifier (VE-AN; BMI ≤ 13.5); and (c) to verify inpatients’ hospitalization outcome according to BMI severity. We enrolled 168 inpatients with AN completing the following: Eating disorder Examination-Questionnaire, Eating Disorder Inventory-2, State-Trait Anxiety Inventory, Beck Depression Inventory, Body Shape Questionnaire, and EQ-5D-VAS. According to the current BMI classification, those with a BMI < 15 versus those with non-extreme AN (NE-AN, BMI ≥ 15) differed on all measures but the quality of life with those with NE-AN reporting more impaired scores on all measures. Adopting an exploratory classification comparing VE-AN, extreme AN (E-AN, BMI = 13.6–14.99), and NE-AN, no differences emerged between VE-AN and E-AN, while those with NE-AN reported significantly more impaired scores on all variables while the quality of life again did not differ across groups. Hospitalization outcome improved for all groups, independently of BMI. Groups differed concerning the length of stay that mirrored BMI severity and impacted also hospitalization outcomes. Taken together, our data support the lack of validity of current BMI specifiers in AN, even in the acute setting. Moreover, the exploratory subgroup of patients with BMI ≤ 13.5 did not delineate a clinically different group.

## 1. Introduction

Defining severity in anorexia nervosa (AN) is a significant challenge, therefore in 2013, the DSM-5 [1]—in line with the new specifiers for mental disorders—introduced body mass index (BMI) specifiers, including mild (i.e., BMI ≥ 17), moderate (i.e., BMI between 16 and 16.9), severe (i.e., BMI between 15 and 15.9), and extreme (BMI < 15). However, the available evidence on BMI specifiers in AN has consistently questioned the reliability and the clinical validity of such a potential definition of severity [2,3,4]. For example, outpatients with extreme AN tend not to differ with respect to duration of illness or other clinical characteristics [4,5,6], but have been reported as more frequently diagnosed with the restricting subtype of AN (AN-R) [4,7]. Most importantly, when compared to patients with mild and moderate AN, research consistently showed that those with extreme AN did not differ from those patients with higher BMI on eating psychopathology, as measured by the Eating Disorder Examination-Questionnaire (EDE-Q) [4,6,8], or even showed less marked scores on the same measure [5,7]. Only Dakanalis and colleagues [9] found that BMI specifiers could usefully define eating psychopathology (as measured with the Eating Disorder Examination), perfectionism, self-esteem, and illness-specific functional impairment. However, groups with different BMI specifiers did not differ in comorbidity (anxiety, mood, substance use disorders) and distress [9].

Nevertheless, it is well-known that an intensification of treatment is needed when patients’ BMI is low or decreases rapidly; as a result, a substantial proportion of patients require hospitalization in a very malnutrition-related acute phase of their illness [10]. Therefore, research on outpatients may not apply to the inpatient setting. People with AN who need to be hospitalized can be commonly found in everyday clinical practice and require specific and intensive clinical attention [11]. In this substantial group of patients, data on BMI specifiers are currently particularly sparse and described only by a few studies. Gianini and colleagues [2] found that inpatients with extreme AN reported more previous hospitalizations and a longer duration of illness than those with less severe BMIs. However, across groups with different BMIs, eating psychopathology, as measured by the EDE-Q, was comparable, as were depressive symptoms and quality of life [2]. Along these lines, Dalle Grave [3] found no baseline differences on the EDE-Q and no outcome differences after enhanced cognitive-behavior therapy for inpatients across groups with different BMIs. These few data on AN inpatients seem to preliminarily confirm the lack of reliability of the BMI as a specifier even in the hospital setting. However, people who are hospitalized for AN often have an extreme BMI, but with a wide range, potentially varying from the 15 threshold to very extreme values, even below 10. This broad heterogeneity highlights the need to garner further evidence on this topic, suggesting that the extreme specifier could be investigated more. In fact, it is questionable that the degree of severity can change from mild to extreme due to one point of BMI; however, the extreme category is broad, eventually encompassing several points of BMI (e.g., 9–15). Therefore, the extreme specifier applies equally to people who are in a life-threatening condition, unable to handle everyday activities [12] and patients in a much less severe clinical condition, without functional impairments. The hospitalization setting could be useful as a preferential observation point for those patients with extreme AN who are referred to a hospital facility frequently due to an emergency related to several potentially deadly emaciation-related conditions. Currently, no studies have explored the possibility that the extreme BMI specifier could be somehow specified further. In the case of hospitalization, BMI is frequently much lower than 15, so we became interested in investigating a putative “very extreme” specifier (i.e., BMI ≤ 13.5, according to the 50th percentile in our sample).

Given the aforementioned dearth of studies on BMI specifiers validity for inpatients (even fewer in case of inpatients who need an emergency hospitalization) and the lack of data on a fine-grained analysis of extreme AN, we designed this study with a three-fold aim: (a) to investigate if, even in the context of acute hospitalization, the current extreme BMI specifier (i.e., Current E-AN, BMI < 15) could mirror patients’ clinical severity when compared to those with non-extreme AN (i.e., NE-AN; BMI ≥ 15), as measured by eating and general psychopathology, body image concerns, and quality of life; (b) to explore a novel specifier defining a group of patients with “very extreme” AN (i.e., VE-AN; BMI ≤ 13.5), versus patients with extreme AN (i.e., E-AN; 13.6–14.9) versus patients with NE-AN to ascertain eventual more strict criteria for the current extreme category; and (c) to test if the aforementioned groups could respond differently to the acute hospitalization. We expected that patients in the very extreme AN group could report more severe eating and general symptomatology because of their life-threatening clinical condition.

## 2. Materials and Methods

### 2.1. Participants

Overall, 193 candidates for this study were contacted among those seeking voluntary hospitalization at the Eating Disorders Center of the “Città della Salute e della Scienza” hospital at the University of Turin, Italy from December 2016 to July 2021. The inclusion criteria were as follows: (a) diagnosis of AN as assessed by an experienced psychiatrist with the Structured Clinical Interview for Diagnostic and Statistical Manual of Mental Disorders, 5th edition (DSM-5) [13], (b) age > 18 years old, and (c) no psychotic or bipolar disorders. Of all candidates, 16 returned incomplete assessments, and nine refused study participation. Finally, a total of 168 inpatients with AN were enrolled in this study that was approved by the Ethical Committee of the “Città della Salute e della Scienza” hospital at the University of Turin, Italy, with protocol number 0073951.

### 2.2. Treatment

Details of the treatment can be found elsewhere [10]. Treatment is delivered following the requirements and specificities needed when dealing with patients with AN who need inpatient treatment, therefore the clinical team is multidisciplinary and individualized treatment plans are provided. The majority of patients (over 80%) were admitted through the emergency room in a very acute phase of AN. The intervention is focused on re-establishing patients’ clinical life-threatening conditions, enhancing patients’ motivation concerning the subsequent therapeutic steps. Psychoeducation for families is provided.

### 2.3. Materials

Patients’ sociodemographic and clinical characteristics were collected upon hospital admission (T0) and discharge (e.g., end of treatment, EOT) with a clinical interview. BMI was obtained by a trained nurse after the measurement of patients’ height and weight at both time points.

All participants completed the following assessments at both T0 and EOT:

- Eating disorder Examination Questionnaire (EDE-Q), Italian version [14], assessing eating behaviors that occurred in the last 28 days. It comprehends 28 items, four subscales (dietary restraint, eating concerns, weight concerns, and shape concerns), and a final global score. This tool showed sound psychometric properties with a Cronbach’s alpha in our sample of 0.95.
- Eating Disorder Inventory-2 (EDI-2), Italian version [15], showed a Cronbach’s alpha in our sample of 0.96. It evaluates eating psychopathology via measuring typical eating-disordered behaviors. We focused on three subscales assessing the “core” aspects of eating disorders: drive for thinness (DT), bulimia (B), and body dissatisfaction (BD).
- State-Trait Anxiety Inventory (STAI) [16], consisting of 20 questions about the level of anxiety experienced in the present moment and 20 questions about anxiety conceived as a persistent trait. In our sample, Cronbach’s alpha was 0.96.
- Beck Depression Inventory (BDI) [17], concerning the severity of depressive symptoms expressed by a global score and classified from low to severe with strong internal consistency (Cronbach’s alpha value 0.86) [18]. Cronbach’s alpha in our sample was 0.884.
- Body Shape Questionnaire (BSQ) [19], investigating body image focusing on the last weeks with good psychometric properties [20] (Cronbach’s alpha in our sample 0.96). Higher scores correspond to more severe levels of body dissatisfaction.
- EQ-5D [21], measuring the quality of life and used in multiple fields of medicine with robust psychometric properties [22]. Cronbach’s alpha in our sample was 0.707. It consists of a descriptive five-dimension part regarding mobility, self-care, usual activities, pain/discomfort, and anxiety/depression, plus a visual analog scale (VAS), which was the object of our research, expressing a self-rated measurement of health from 0 to 100.

### 2.4. Statistical Analysis

To compute the analysis, the SPSS 27.0 statistical software package (IBM SPSS Statistics for Windows, Version 27.0. Armonk, NY, USA: IBM Corp.) was used.

Independent sample *t*-test and Fisher’s exact test have been used to compare Current-E-AN and NE-AN groups for continuous and categorical variables, respectively. Cohen’s d effect sizes were calculated as well; differences are defined as small (≥0.2 and <0.5), medium (≥0.5 and <0.8), large (≥0.8) [23].

In order to investigate eventual severity thresholds below BMI = 15, we split the subsample of the patients with BMI < 15 according to the 50° percentile corresponding to 13.5 in our sample. Therefore, three BMI groups were considered for the second and third aims of this study: non-extreme BMI (NE-AN) in the case of BMI ≥ 15; extreme BMI (E-AN) in the case of BMI between 13.6 and 14.9, and very extreme BMI (VE-AN) in the case of BMI ≤ 13.5. One-way ANOVA with eta-squared (η^2^) calculation and Bonferroni post-hoc analysis and Fisher’s exact test were run to compare the aforementioned three groups. Differences were estimated as small η^2^ = 0.01–0.05; moderate η^2^ = 0.06–0.14; and large η^2^ > 0.14 [23]. Analysis of covariance was run to control all comparisons for the diagnostic subtype of AN.

Repeated measure ANOVA with eta-squared (η^2^) calculation has been run to verify the eventual difference in clinical outcome (i.e., BMI, EDE-Q total score, EDI-2 “core” subscales, depressive and anxiety symptoms, body image dissatisfaction, and quality of life) across the three groups with different severity of BMI during hospitalization.

## 3. Results

### 3.1. Sociodemographic and Clinical Characteristics of the Sample

Patients were all Caucasian and voluntarily admitted. None of them left the program against medical advice. Of 168 patients, nine were males (5.4%);125 participants (74.4%) were diagnosed with the restricting (AN-R), while 43 (25.6%) with the binge-purging (AN-BP) subtype of AN. The mean age of the sample was 24.3 ± 9.5 years (range: 18–56 years), the mean duration of illness was 6.1 ± 7.8 years (range: 0.5–40 years), and the mean BMI was 14.3 ± 1.9. Overall, 73 patients (43.5%) reported previous hospitalizations and the mean duration of hospitalization was 35.3 ± 14.8 days.

When comparing patients with Current-E-AN versus those with NE-AN, no differences emerged with respect to sex and previous hospitalizations (data not shown) but those in the Current-E-AN group were more frequently diagnosed with AN-R than those with NE-AN (Current-E-AN: *n* = 91 (72.8%); NE-AN *n* = 34 (27.2%), Fisher’s exact test *p* = 0.003).

Concerning the comparison of the VE-AN, E-AN, and NE-AN groups, those in the VE-AN group were more frequently diagnosed with AN-R when compared to the other groups (VE-AN: *n* = 55 (44%); E-AN *n* = 36 (28.8%); NE-AN *n* = 34 (27.2%), Fisher’s exact test *p* = 0.001). No differences emerged across the three BMI severity groups regarding sex (VE-AN: *n* = 2 (22.2%); E-AN *n* = 5 (55.6%); NE-AN *n* = 2 (22.2%), Fisher’s exact test *p* = 0.261) and previous hospitalizations (VE-AN: *n* = 23 (31.5%); E-AN *n* = 25 (34.2%); NE-AN *n* = 25 (34.2%), Fisher’s exact test *p* = 0.346).

### 3.2. Comparison between Groups of BMI Severity According to the Current Classification

No differences emerged when comparing Current-E-AN and NE-AN concerning age and duration of illness, while the groups significantly differed in length of stay (see Table 1).

As shown in Table 1, Current-E-AN and NE-AN differed on all considered variables, with the exception of the quality of life. In particular, those in the NE-AN group reported more severe scores than those in the Current-E-AN group on all the significantly different assessments with medium to large effect sizes (see Table 1).

### 3.3. Comparison across Groups of BMI Severity According to the Novel Exploratory Classification

As shown in Table 2, when comparing VE-AN, E-AN, and NE-AN groups no differences concerning age or duration of illness emerged, while an overall difference in length of hospitalization emerged. The post-hoc analysis showed that VE-AN and E-AN had a comparable length of stay, as well as E-AN and NE-AN, although a significant difference emerged between VE-AN and NE-AN.

When comparing VE-AN, E-AN, and NE-AN groups, those in the VE-AN and E-AN groups reported similar scores on eating psychopathology (see Table 2). With more detail, the E-AN group reported intermediate scores (although not statistically different from those of VE-AN) while the NE-AN reported the most severe ones. The same trend was found for trait anxiety, depressive symptoms, and body image concerns. That is, the VE-AN and E-AN groups reported comparably lower scores when compared to the NE-AN group with effect sizes ranging from medium to large (see Table 2). Symptoms of state anxiety reported less marked differences: only the VE-AN versus NE-AN comparison resulted to be significantly different.

With respect to the investigation of clinical outcome across the VE-AN, E-AN, and NE-AN groups (see Table 3), we found that all patients significantly improved on all measures considered but body dissatisfaction, as measured by the EDI-2, during hospitalization. Moreover, all groups differed in baseline scores with the exception of quality of life, in line with the aforementioned (see Table 1 and Table 2) data. Importantly, the lack of significant time*group interactions showed that all groups showed similar trajectories of improvement, if any (see Table 3). In contrast, the only significant time*group interaction was found for change in BMI during hospitalization, with those in the VE-AN group showing the greatest improvement in BMI, as measured upon discharge.

Since the three groups showed significantly different lengths of stay, we controlled our analyses for such a variable. After statistical control, EDI-2 drive for thinness, trait anxiety, body image concerns, and quality of life did not survive significance, thus resulting as time-dependent outcomes of hospitalization (see Table 3).

## 4. Discussion

With this study, we aimed to investigate both current and innovative BMI specifiers for AN in the context of acute hospitalization, with the focus on expanding knowledge on a potential “very extreme” (BMI ≤ 13.5) specifier for AN. Three main findings emerged from this study: first, comparing patients with extreme and non-extreme AN, according to the current DSM-5 criteria, the latter group showed comparable quality of life but significantly more severe eating and general psychopathology, coupled with more marked body image concerns than their counterpart. Second, the exploration of a further specification for the current extreme AN category with a group of patients with very extreme AN (i.e., VE-AN, BMI ≤ 13.5) yielded a comparable trend: namely, those in the VE-AN group—even in the face of a much life-threatening BMI requiring a significantly longer length of stay—scored similarly to the E-AN group, but reported less severe scores on eating psychopathology, anxiety and depressive symptoms, and body image concerns when compared to those with NE-AN. In contrast, the three groups did not differ in quality of life. Finally, all three groups responded equally well to the acute inpatient treatment, with the length of stay significantly impacting clinical outcome.

Patients with AN tend to require hospitalization in a substantial number of cases and such an intensification of care can be required for serious and strictly AN-related conditions (i.e., electrolyte abnormalities, bradycardia, total fasting). In this context, BMI specifiers of AN have been poorly investigated, notwithstanding the need for reliable and clinically meaningful parameters for defining severity, even in such an acute context. Taken together, our findings on the comparison between extreme and non-extreme groups of patients with AN, according to the current BMI specifiers, were overall in line with earlier literature that questioned the validity of BMI specifiers as a proxy for patients’ clinical severity [4,5,7]. However, in contrast to earlier research which does not report differences across groups [4], our findings are more in-keeping with studies on outpatients [5,7]. In fact, we found patients with extreme AN reporting less severe psychopathology than their counterparts. The Current-E-AN group reported less severe eating psychopathology, anxiety and depressive symptoms, and body image concerns than those with greater BMI. Only quality of life did not differ across groups, potentially due to the life-threatening condition that determined for all patients the hospitalization itself. As a novel contribution to the literature, those in the Current-E-AN groups showed a significantly longer length of stay than those with NE-AN.

Moreover, prompted by our clinical experience with severely emaciated patients with AN, we explored a novel “very extreme” BMI specifier including patients with a BMI ≤ 13.5 since, to the best of our knowledge, this kind of investigation has not been performed so far. Interestingly, those in the VE-AN group were more frequently diagnosed with AN-R, in line with earlier data on patients with extreme AN [4,7] but, overall, the three groups were comparable for sex and duration of illness, in contrast with earlier work [7]. Unexpectedly, and in contrast with our a priori hypothesis, the VE-AN group reported the mildest scores, when compared to the other groups with greater BMIs, on eating psychopathology. Moreover, it is noteworthy that no statistical differences could be found between VE-AN and E-AN groups, overall supporting the current specifier threshold. This trend of marked psychopathology in non-emaciated patients resembles only one study on outpatients [7], but is not in keeping with the available reports on inpatients that reported no differences across groups with different BMIs on the EDE-Q [2,3]. This conflicting result (low BMI coupled with mild EDE-Q scores) may be due to patients’ potential lack of awareness of their severe condition. It is well-known that AN is an ego-syntonic disorder [24], therefore those with a very extreme BMI could tend to minimize their overall severity, even more than those patients with higher BMIs. In this case, the very extreme degree of emaciation could pathologically “mitigate” the cognitive symptoms of AN by constituting a powerful maintenance factor that cognitive models describe as pro-ANA beliefs [25]. Further studies adding a clinician-based evaluation could clarify this issue. Similarly, the EDI-2 data did not help define severity in our sample; although drive for thinness has been proposed as a reliable marker of severity [10,26], it failed to discriminate those in the VE-AN or E-AN group. In contrast, it is noteworthy that dissatisfaction for body image has not been measured in the past, thus our data are difficult to compare; however, patients’ marked emaciation could contribute to explain the lower levels of dissatisfaction for body image concerns reported by those with VE-AN and E-AN when compared to patients with NE-AN. Additionally, patients in the VE-AN and E-AN groups were less depressed than those patients with higher BMI. Again, the few available studies reported a lack of differences on the BDI [2,5], while we found a trend overall comparable to that of eating psychopathology. With respect to anxiety, trait anxiety reported the same trend found on all previous assessments while state anxiety resulted to be more comparable across groups, with only one significantly different comparison between VE-AN and NE-AN. It is noteworthy that these data survived significance also after statistical control for the diagnostic subtype. This is of interest, since patients with AN-BP are proposed as more severe from a psychopathology standpoint [27,28]. Finally, the lack of statistical differences in quality of life could reflect patients’ severe and acute overall clinical condition upon admission, which could level out patients’ scores on this assessment. It is noteworthy that, similarly to the comparison between patients with Current-E-AN and NE-AN, the use of a novel exploratory classification also confirmed a statistically significant difference across the three groups of BMI severity on the duration of hospitalization. This is of interest as, in spite of the self-reported evaluations of psychopathology, length of stay seems to represent a rather objective proxy for patients’ severity, resonating with BMI. However, it is noteworthy that VE-AN and E-AN groups showed an overall comparable length of stay, also questioning in this regard the validity of the VE-AN threshold. Nevertheless, since no earlier studies investigated this aspect, this is a novel finding requiring further investigations.

Interestingly, independently of the BMI severity, patients in the VE-AN, E-AN, and NE-AN groups improved during the hospital stay on all outcomes considered, namely eating psychopathology, state anxiety, depressive symptoms, and body image concerns. These are innovative data because our sample included patients who required an emergency hospitalization, thus those particularly severe and acutely ill. Additionally, our finding resonates with the only other available study on this topic [3] that, however, relied on patients with comparable severity across groups. In fact, Dalle Grave and collaborators [3] did not report significant baseline differences on the EDE-Q across groups with different BMIs. It should be highlighted that length of stay had an impact on hospitalization trajectory; after statistical control for this parameter, EDI-2 drive for thinness, trait anxiety, body image concerns, and quality of life resulted as not significantly improved upon discharge suggesting how the latter measures tend to need a longer timeframe to improve. Earlier research suggested drive for thinness as more relevant for framing severity in AN [26], thus it is reasonable that more time can be required to improve such a critical—also with respect to outcome [10]—measure. Moreover, patients’ anxiety is a hallmark of AN even after recovery [29], and earlier research from our group showed that several components of body image are at play during hospitalization [30], therefore duration of hospital stay can influence the outcome concerning these measures. Finally, it is well-known that quality of life is particularly impaired in inpatients with AN—it has been scored as even worse than death [31]—thus once more duration of treatment can be of utmost importance in this regard. Notwithstanding, taken together, our results question the overall validity of BMI specifiers not only as severity indices but also as prognostic factors of treatment response after hospitalization. With more detail, BMI could be a generic proxy for organic severity, mostly for highly emaciated patients as those highlighted by our data but that could paradoxically not be described by the current DSM-5 classification. However, BMI resulted not be a valid index for psychopathology and patients’ subjective perceived suffering. The data on the VE-AN group suggest that it would be beneficial to include also patients’ degree of insight of AN, in line with the specifiers for Obsessive-Compulsive Disorder (e.g., good or fair, poor, and absent insight; DSM-5 [1]). In fact, it is possible that some patients with VE-AN could not recognize their critical condition.

Despite some strengths including a real-world setting, the lack of insurance barriers, and the comprehensive assessment of several clinically relevant parameters, this study suffers from limitations as well: inpatients with severe AN were included, potentially jeopardizing data generalizability, mostly concerning their psychopathological conditions requiring emergency hospitalization. Moreover, this type of analysis can be biased as follows: patients with higher BMI could be more critical from a general psychopathology perspective (i.e., suicidal thoughts, impulsivity, severe purging behaviors, severe cognitive eating symptoms), while patients with low BMI could report an overall acceptable clinical condition. With that being said, we reiterate that BMI is a highly somatic index, unable to fully capture the severity of AN which includes much more complex aspects like psychopathology, emaciation, illness denial, proneness to collaboration.

## 5. Conclusions

In closing, our data do not support the validity of current BMI specifiers in the definition of severity for inpatients with severe AN. Moreover, we did not find that the delineation of a subgroup of patients with extremely low BMIs (i.e., <13.5) fruitfully differentiated groups of patients with different extents of clinical severity, since the very extreme and extreme groups showed overall comparable scores on all assessments and similar duration of hospitalization as well, as a proxy for their organic clinical severity. Moreover, even in the face of extremely severe emaciation, our data highlighted how all patients could be able to improve their baseline condition with hospitalization, with similar trajectories notwithstanding their BMI. Given the clinical need for finding reliable specifiers for the severity of AN, other options—including an insight specifier—should be considered.

## Figures and Tables

**Table 1:** Comparison between groups of patients with anorexia nervosa with different body mass index severity according to the current classification.

Total Sample of Inpatients with AN *n* = 168					
	Current Extreme AN (Current-E-AN) BMI < 15 *n* = 111	Non-Extreme AN (NE-AN) BMI ≥ 15 *n* = 57	Test Statistics		
	Mean (SD)	Mean (SD)	*t*	*p*	Cohen’s d
**Age, years**	24 (9)	23.7 (8.4)	0.233	0.816	0.04
**Duration of illness, years**	6.1 (7.6)	5.7 (7.9)	0.375	0.708	0.06
**Duration of hospitalization, days**	37.5 (19)	30.3 (13.8)	2.638	**0.009**	0.43
**EDE-Q**					
Restraint	2.7 (2.1)	4.2 (1.5)	4.79	<0.001 *	0.78
Eating concern	2.5 (1.7)	3.8 (1.2)	4.88	<0.001 *	0.80
Shape concern	3.6 (1.8)	4.8 (1.2)	4.74	<0.001 *	0.78
Weight concern	3.1 (1.8)	4.4 (1.5)	4.48	<0.001 *	0.76
Total score	2.9 (1.7)	4.3 (1.2)	5.26	<0.001 *	0.86
**EDI-2**					
Drive for Thinness	10.3 (5.8)	16.1 (5.8)	4.79	<0.001 *	0.80
Bulimia	1.9 (3.8)	5.3 (5.6)	4.55	<0.001 *	0.76
Body Dissatisfaction	12.8 (6.5)	16.6 (6.6)	3.45	0.001 *	0.56
**STAI**					
State	52.3 (14.5)	58.8 (12.3)	2.81	0.005 *	0.47
Trait	54.5 (12.8)	63.9 (12.7)	4.39	<0.001 *	0.73
**BDI**	13.7 (7.5)	20.7 (7.3)	5.63	<0.001 *	0.93
**BSQ**	105.3 (43.6)	142 (38)	5.16	<0.001 *	0.88
**EQ-5D-VAS**					
VAS	51 (22.4)	46.2 (25.5)	1.15	0.249	-
Index	0.5 (0.5)	0.4 (0.4)	1.31	0.193	-

Bold text indicates statistical significance. * Significant after correction for the diagnostic subtype of anorexia nervosa. Cohen’s d effect sizes: small: >0.2 and <0.5, medium: >0.5 and <0.8, large: ≥0.8. Legend: AN = anorexia nervosa; BMI = body mass index; EDE-Q = Eating Disorder Examination Questionnaire; EDI-2 = Eating Disorders Inventory-2; BDI = Beck Depression Inventory; STAI-T = State-Trait Anxiety Inventory-Trait; STAI-S = State-Trait Anxiety Inventory-State; BSQ = Body Shape Questionnaire; EQ-5D-VAS: EuroQoL Health Questionnaire/Visual Analogue Scale.

**Table 2:** Comparison across groups of patients with anorexia nervosa with different body mass index severity according to the novel exploratory classification.

Total Sample of Inpatients with AN *n* = 168							
	Very Extreme AN BMI ≤ 13.5 (VE-AN) *n* = 62	Extreme AN BMI = 13.6–14.9 (E-AN) *n* = 49	Non-Extreme AN BMI ≥ 15 (NE-AN) *n* = 57	Test Statistics			
	Mean (SD)	Mean (SD)	Mean (SD)	F	*p*	η^2^	Bonferroni Post-Hoc Test
**Age, years**	24.3 (9.5)	23.6 (8.6)	23.7 (8.4)	0.125	0.882	0.002	-
**Duration of illness, years**	6.1 (7.8)	6.2 (7.5)	5.7 (7.9)	0.073	0.930	0.001	-
**Duration of hospitalization, days**	40.4 (21.7)	33.8 (10.9)	30.3 (13.8)	5.711	0.004 *	0.06	VE-AN = E-AN E-AN = NE-AN VE-AN > NE-AN
**EDE-Q**							
Restraint	2.5 (2.1)	2.9 (2.1)	4.2 (1.5)	11.790	<0.001 *	0.13	VE-AN = E-AN < NE-AN
Eating concern	2.5 (1.8)	2.6 (1.5)	3.8 (1.2)	11.926	<0.001 *	0.13	VE-AN = E-AN < NE-AN
Shape concern	3.3 (1.7)	3.9 (1.8)	4.8 (1.2)	13.635	<0.001 *	0.14	VE-AN = E-AN < NE-AN
Weight concern	2.9 (1.8)	3.4 (1.8)	4.4 (1.5)	11.426	<0.001 *	0.12	VE-AN = E-AN < NE-AN
Total score	2.8 (1.7)	3.2 (1.6)	4.3 (1.2)	15.073	<0.001 *	0.16	VE-AN = E-AN < NE-AN
**EDI-2**							
Drive for Thinness	9.8 (8.1)	10.9 (7.7)	16.1 (5.8)	11.796	<0.001 *	0.13	VE-AN = E-AN < NE-AN
Bulimia	1.6 (3.3)	2.3 (4.4)	5.3 (5.6)	10.665	<0.001 *	0.12	VE-AN = E-AN < NE-AN
Body Dissatisfaction	12.5 (6.2)	13.4 (6.9)	16.6 (6.6)	6.224	0.003 *	0.07	VE-AN = E-AN < NE-AN
**STAI**							
State	51 (14.8)	54 (14.1)	58.8 (12.3)	4.582	0.012 *	0.05	VE-AN = E-AN; E-AN = NE-AN; VE-AN < NE-AN
Trait	53.9 (13.8)	55.4 (11.7)	63.9 (12.7)	9.822	<0.001 *	0.11	VE-AN = E-AN < NE-AN
**BDI**	13.5 (7.8)	14 (7.2)	20.7 (7.3)	15.853	<0.001 *	0.17	VE-AN = E-AN < NE-AN
**BSQ**	98.8 (43.7)	114.3 (42.3)	142 (38.1)	15.272	<0.001 *	0.17	VE-AN = E-AN < NE-AN
**EQ-5D-VAS**							
VAS	48.8 (22.5)	54 (22.2)	46.2 (25.5)	1.286	0.279	-	-
Index	0.5 (0.6)	0.5 (0.4)	0.4 (0.4)	0.915	0.403	-	-

Bold text indicates statistical significance. * Significant after correction for the diagnostic subtype of anorexia nervosa. Magnitude of the effect: η^2^ = 0.01–0.05 small; η^2^ = 0.06–0.14 moderate; η^2^ > 0.14 large. Legend: AN = anorexia nervosa; BMI = body mass index; EDE-Q = Eating Disorder Examination Questionnaire; EDI-2 = Eating Disorders Inventory-2; BDI = Beck Depression Inventory; STAI-T = State-Trait Anxiety Inventory-Trait; STAI-S = State-Trait Anxiety Inventory-State; BSQ = Body Shape Questionnaire; EQ-5D-VAS: EuroQoL Health Questionnaire/Visual Analogue Scale.

**Table 3:** Clinical outcome of hospitalization across groups with different body mass index severity.

Total Sample of Inpatients with AN *n* = 168															
	Very Extreme AN BMI ≤ 13.5 (VE-AN) *n* = 62		Extreme AN BMI = 13.6–14.9 (E-AN) *n* = 49		Non-Extreme AN BMI ≥ 15 (NE-AN) *n* = 57		Test Statistics								
	Mean (SD)	Mean (SD)	Mean (SD)	Mean (SD)	Mean (SD)	Mean (SD)	Main Effect of Group			Time*Group Interaction			Main Effect of Time		
	T0	EOT	T0	EOT	T0	EOT	F	*p* *	η^2^	F	*p* *	η^2^	F	*p* *	η^2^
**BMI**	12.4 (0.8)	13.5 (0.9)	14.2 (0.4)	15 (0.7)	16.4 (1.6)	16.8 (1.4)	239.178	**<0.001**	0.746	14.419	<0.001	0.150	152.705	**<0.001**	0.484
**EDE-Q**															
Restraint	2.5 (2.1)	1.6(1.6)	2.9 (2.1)	1.8(1.5)	4.2 (1.5)	3(1.8)	10.946	**<0.001**	0.153	0.201	0.819	0.003	72.601	**<0.001**	0.375
Eating concern	2.5 (1.8)	1.8 (1.5)	2.6 (1.5)	2.1 (1.4)	3.8 (1.2)	3.1 (1.3)	7.873	**0.001**	0.115	0.239	0.788	0.004	33.765	**<0.001**	0.218
Shape concern	3.3 (1.7)	2.8 (1.9)	3.9 (1.8)	3.23 (1.8)	4.8 (1.2)	4.1 (1.8)	7.110	**0.001**	0.105	0.445	0.642	0.007	33.938	**<0.001**	0.219
Weight concern	2.9 (1.8)	2.4 (1.8)	3.4 (1.8)	2.6 (1.7)	4.4 (1.5)	3.5 (1.9)	7.253	**0.001**	0.107	1.133	0.325	0.018	40.766	**<0.001**	0.252
Total score	2.8 (1.7)	2.1 (1.6)	3.2 (1.6)	2.4 (1.5)	4.3 (1.2)	3.4 (1.6)	9.621	**<0.001**	0.137	0.300	0.741	0.005	63.892	**<0.001**	0.346
**EDI-2**															
Drive for Thinness	9.8 (8.1)	9.6 (8.1)	10.9 (7.7)	10.5 (7.7)	16.1 (5.8)	14.7 (7)	5.687	**0.004**	0.091	0.348	0.707	0.006	6.954	**0.01 ^§^**	0.058
Bulimia	1.6 (3.3)	0.9 (1.8)	2.3 (4.4)	1.3 (3.3)	5.3 (5.6)	3.2 (3.1)	7.6	**0.001**	0.119	1.865	0.160	0.032	27.847	**<0.001**	0.198
Body Dissatisfaction	12.5 (6.2)	12.3 (6.5)	13.4 (6.9)	12.9 (7.2)	16.6 (6.6)	16.1 (7..8)	4.043	**0.020**	0.067	0.428	0.653	0.008	2.583	0.111	0.023
**STAI**															
State	51 (14.8)	49.7 (15.7)	54 (14.1)	47.4 (15.8)	58.8 (12.3)	55.2 (13.8)	2.881	**0.060**	0.046	2.782	0.066	0.044	14.368	**<0.001**	0.107
Trait	53.9 (13.8)	52.9 (14.2)	55.4 (11.7)	52 (17.3)	63.9 (12.7)	58.3 (12.1)	4.880	**0.009**	0.075	2.224	0.113	0.036	13.717	**<0.001 ^§^**	0.103
**BDI**	13.5 (7.8)	10.2 (7.8)	14 (7.2)	8.7 (6.6)	20.7 (7.3)	14 (9.2)	6.243	**0.003**	0.097	0.716	0.491	0.012	50.748	**<0.001**	0.304
**BSQ**	98.8 (43.7)	95.8 (44.3)	114.3 (42.3)	102.8 (41.3)	142 (38.1)	129.5 (45.8)	8.163	**<0.001**	0.135	1.312	0.274	0.024	12.862	**<0.001 ^§^**	0.109
**EQ-5D-VAS**															
VAS	48.8 (22.5)	64.8 (18.6)	54 (22.2)	64.4 (22.7)	46.2 (25.5)	49.8 (25.6)	2.349	0.101	0.046	1.406	0.250	0.028	13.641	**<0.001 ^§^**	0.123
Index	0.5 (0.6)	0.7 (0.3)	0.5 (0.4)	0.7 (0.2)	0.4 (0.4)	0.6 (0.3)	0.888	0.414	0.016	0.099	0.906	0.002	16.206	**<0.001 ^§^**	0.131

Bold text indicates statistical significance. * The significance value of all *p* values did not change after statistical correction for length of hospitalization, with the only exceptions highlighted with ^§^. ^§^ No longer significant after statistical correction for length of hospitalization. Magnitude of the effect: η^2^ = 0.01–0.05 small; η^2^ = 0.06–0.14 moderate; η^2^ > 0.14 large. Legend: AN = anorexia nervosa; BMI = body mass index; EDE-Q = Eating Disorder Examination Questionnaire; EDI-2 = Eating Disorders Inventory-2; BDI = Beck Depression Inventory; STAI-T = State-Trait Anxiety Inventory-Trait; STAI-S = State-Trait Anxiety Inventory-State; BSQ = Body Shape Questionnaire; EQ-5D-VAS: EuroQoL Health Questionnaire/Visual Analogue Scale.

## Data Availability

Anonymized data can be required to the last author upon reasonable request.
